# Circulating Thyroid Hormone Profile in Response to a Triiodothyronine Challenge in Familial Longevity

**DOI:** 10.1210/jendso/bvaa117

**Published:** 2020-08-20

**Authors:** Ana Zutinic, Gerard J Blauw, Hanno Pijl, Bart E Ballieux, Rudi G J Westendorp, Ferdinand Roelfsema, Diana van Heemst

**Affiliations:** 1 Department of Internal Medicine, Division of Gerontology and Geriatrics, Leiden University Medical Centre, Leiden, ZA, the Netherlands; 2 Department of Internal Medicine, Division of Endocrinology and Metabolic Diseases, Leiden University Medical Centre, Leiden, the Netherlands; 3 Department of Clinical Chemistry and Laboratory Medicine, Leiden University Medical Centre, Leiden, the Netherlands; 4 Public Health and Centre for Healthy Aging, University of Copenhagen, Copenhagen, Denmark

**Keywords:** thyroid, TSH, 3,5,3′-triiodothyronine, longevity, negative feedback

## Abstract

**Context:**

Familial longevity is associated with higher circulating levels of thyrotropin (TSH), in the absence of differences in circulating thyroid hormones, and a lower thyroid responsivity to TSH, as previously observed in the Leiden Longevity Study (LLS). Further mechanisms underlying these observations remain unknown.

**Objective:**

We hypothesized that members from long-lived families (offspring) have higher thyroid hormone turnover or less negative feedback effect on TSH secretion compared to controls.

**Methods:**

In a case-control intervention study, 14 offspring and 13 similarly aged controls received 100 µg 3,5,3′-triiodothyronine (T3) orally. Their circulating T3, free T3 (fT3), and TSH levels were measured during 5 consecutive days. We compared profiles of circulating T3, fT3, and TSH between offspring and controls using general linear modeling (GLM) and calculated the percentage decline in TSH following T3 administration.

**Results:**

Circulating T3 and fT3 levels increased to supraphysiologic values and normalized over the course of 5 days. There were no serious adverse events. T3 and fT3 concentration profiles over 5 days were similar between offspring and controls (T3 GLM *P* = .11, fT3 GLM *P* = .46). TSH levels decreased in a biphasic manner and started returning to baseline by day 5. The TSH concentration profile over 5 days was similar between offspring and controls (GLM *P* = .08), as was the relative TSH decline (%).

**Conclusions:**

Members of long-lived families have neither higher T3 turnover nor diminished negative feedback of T3 on TSH secretion. The cause and biological role of elevated TSH levels in familial longevity remain to be elucidated.

Thyrotropin (TSH) levels tend to increase with age [1]. It is unknown to what extent this increase reflects selective survival of people with a genetic predisposition for relatively higher TSH [2], and to what extent it reflects an adaptive process.

In the Leiden Longevity Study (LLS) it has previously been observed that members of long-lived families (offspring) had on average 0.8 mU/L higher TSH levels than similarly aged controls throughout a 24-hour period, whereas thyroid hormones were not different between the groups [3]. This discrepancy could not be explained by a reduced bioactivity of TSH, which has been shown to not differ between offspring and controls [3].

This study is 1 of 2 studies we performed in parallel to further understand the mechanisms behind higher TSH in offspring than controls in the presence of similar thyroid hormone levels. We have recently shown that offspring have lower thyroidal response to TSH by performing a challenge study with recombinant human TSH [4]. We hypothesized that offspring might also have higher turnover of thyroid hormones and/or less negative feedback on TSH.

To investigate this, we performed a single challenge study with 100 µg 3,5,3′-triiodothyronine (T3) in offspring from long-lived families and controls. We were not expecting complete inactivating receptor defects of the thyroid hormone receptor or activating mutations of deiodination enzymes or enzymes of the sulphating pathways, but rather subtle defects as found in polymorphisms. We hypothesized that on administration of a single dose of T3, offspring would have a higher decrease in concentration profiles of circulating T3 and free T3 (fT3) than controls and less negative feedback of T3 on TSH.

We believe that these mechanisms are not mutually exclusive but could in concert be contributing to the previously observed differences in thyroid axis phenotype between offspring and controls.

## 1. Materials and Methods

### A. Study Population

The LLS was founded in 2002 and designed to investigate genotypes and phenotypes underlying interindividual differences in familial longevity in humans [5]. Individuals recruited into the study were siblings from 421 Caucasian long-living families (men aged 89 and older, women aged 91 and older) living in the Netherlands in the early 2000s, without any restrictions on health or demographics [6]. The offspring of these families, who were also found to have a lower morbidity than controls [6], were also asked to participate in the study, with their current partners as a reference group, thereby creating a case group enriched for longevity (offspring) and a reference group with similar lifestyle factors and socioeconomic status as the case group, but without selection for familial predisposition to longevity (controls).

Individuals were recruited for the T3 challenge study from the subgroup of LLS previously studied in terms of thyroidal status between offspring and controls [3], and excluded based on laboratory results (hemoglobin < 7.1 mmol/L, TSH > 4.0 mU/L, free thyroxine [fT4] < 9 pmol/L or > 24 pmol/L, thyroid peroxidase [TPO] antibody positivity > 35 kU/L), medical history (cardiac arrhythmias, [history of] thyroid diseases, renal, hepatic or endocrine disease, or any other significant chronic disease), medication use (hormone therapy, thyroid medication), lifestyle factors (nicotine abuse, [history of] alcohol abuse [> 28 units/week], and practical factors (difficulty inserting an intravenous cannula, participation in other research projects within the last 3 months, participation in 2 or more projects in 1 year, evaluation by physician as too vulnerable to participate). Our participants had no palpable goiter.

### B. Clinical Protocol

Participants were admitted into the study after passing the medical screening. The study consisted of 5 consecutive study days at Leiden University Medical Centre. On the morning of study day 1, participants were in a fasted state, an intravenous cannula was placed in a forearm vein, blood was withdrawn at baseline, and 100 µg (4 × 25 µg) T3 was administered orally with a glass of water. The time of administration was used as reference, time 0. Thereafter, blood was sampled at high frequency: every 15 minutes in the first 4 hours after administration, every 30 minutes between 4 and 7 hours after T3 administration, and finally every hour between 7 and 9 hours after T3 administration. During this time, participants received 3 standardized meals (after the 2-hour blood sample, 5-hour blood sample, and 8-hour blood sample), each consisting of 600 kcal (2 × 125 mL Nutridrink Compact, Nutricia Advanced Medical Nutrition). On study days 2, 3, 4, and 5, additional fasted blood samples were obtained at respectively 24, 48, 72, and 96 hours after T3 administration. Participants were at their leisure outside these times.

In total, 370 mL of blood was withdrawn from each individuals across 29 time points (25 on study day 1, and 1 on days 2, 3, 4, and 5).

Height, weight, and body composition were measured on study day 2. Body composition was measured with a Bioelectrical Impedance Analysis meter at a fixed frequency of 50 kHz (Bodystat 1500 Ltd [7]).

The study was designed in accordance with the Declaration of Helsinki and has been approved by the medical ethical committee of the Leiden University Medical Centre. It is registered at Leiden University Medical Centre under the protocol P16.107 and with EudraCT under the number 2016-001497-15. All participants gave written informed consent prior to the screening visit.

### C. Handling of Samples

Serum samples were kept at room temperature for 60 minutes to clot before being processed at the Clinical Chemistry and Laboratory Medicine Department, Leiden University Medical Centre. Samples were centrifuged for 10 minutes at 2350 *g* relative centrifugal force at a temperature of 20°C. After being transferred to 500-µL aliquots, serum samples were temporarily stored at –20°C prior to permanent storage at –80°C until analysis.

### D. Laboratory Measurements

Laboratory measurements were performed after all participants had completed the study. We first measured samples from 6 participants as a pilot to identify whether T3 and fT3 were increased and at which time points, followed by measurements of samples from the remaining 21 participants at all time points. All measurements were performed with the same lot number. All samples from one individual were measured in the same batch.

### E. Assays and Assay Performance

Laboratory measurements were performed with fully automated, software-monitored equipment and diagnostics from Roche Diagnostics at the Clinical Chemistry and Laboratory Medicine Department at Leiden University Medical Centre. Aspartate aminotransferase (catalog No. 11876848216), alanine aminotransferase (catalog No. 11876805216), and creatine (catalog No. 5168589190) were measured from a fasted morning serum sample using the Modular P800 clinical chemistry analyzer. Creatine values were used to estimate glomerular filtration rate using the CKD-EPI (Chronic Kidney Disease Epidemiology Collaboration) calculation. Thyroid parameters for TSH [8], fT4 [9], T4 [10], fT3 [11], and T3 [12] were measured in serum by an immunoassay using Roche cobas8000 with an E602 module. The coefficient of variation for TSH was 2.36% (SD 0.52), for fT4 5.55% (SD 2.28), for fT3 2.06% (SD 0.58), for T3 5.25% (SD 0.34).

### F. Statistical Analyses

Descriptive statistics were used to summarize group characteristics. Independent samples *t* test, Mann-Whitney U test, and chi-square test were used, depending on the characteristics of the variable (normally distributed, not normally distributed, and categorical, respectively), to statistically test for differences between offspring and controls regarding demographics, anthropometrics, and laboratory measurements. General linear modeling (GLM) was used to investigate differences in the concentration profiles of T3, fT3, and TSH between offspring and controls. In all analyses, a *P* value of less than or equal to .05 was considered statistically significant.

Previous studies have investigated the effect of T3 administration on thyroid function in healthy individuals. Spencer et al showed that oral administration of a single dose of 100 µg synthetic T3 significantly suppressed TSH levels [13]. It was hypothesized that controls would show the same pattern in T3 and TSH levels after T3 administration as was observed in the study performed by Spencer and colleagues, whereas for the offspring a faster decrease in T3 levels was expected. Consequently, for TSH levels it was hypothesized that offspring would show higher levels compared to controls because of the faster disappearance of T3. Based on the abovementioned assumptions, a sample size calculation was performed to compare the expected area under the curve (AUC) in the TSH suppression curves of offspring and controls. For the sample size calculation, an SD of 30.6 was used, which is 2 times the SD of the 24-hour AUC TSH for the controls as observed in Switchbox [3].

A 2-sided significance level of 5% was used and the power was set at 80%. Based on these assumptions, a sample size of 10 participants in each group was needed to measure a 1.93 times larger TSH AUC in offspring compared to controls after T3 administration with 80% statistical power. To overcome possible dropout due to difficulties with blood sampling or laboratory measurements, we aimed to include 15 participants in each group.

Programs used for statistical analyses were SPPS for Windows, version 23 (SPSS), Systat version 13 (Systat Software, Inc), and Matlab (The MathWorks Inc). Graphs were made using Microsoft Office Excel 2016 and GraphPad Prism for Windows, version 8.1.1 (330) (GraphPad Software, Inc).

## 2. Results

### A. Inclusions

**Figure 1.**
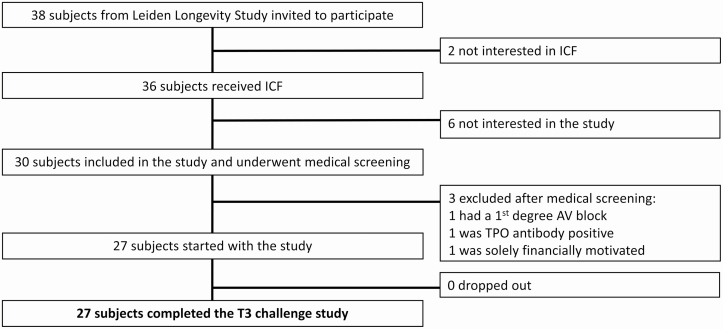
Recruitment and inclusion of participants for the 3,5,3′-triiodothyronine (T3) challenge study. AV, atrioventricular; ICF, informed consent form; TPO, thyroid peroxidase.

The recruitment and inclusion flowchart is presented in Fig. 1. Thirty-eight individuals were invited by telephone to participate. Two of these individuals were not interested in receiving the informed consent form. Out of the 36 individuals who did receive an informed consent form, 6 were not interested in participating in the study. Thirty individuals were included in the study and underwent a medical screening. Three individuals were excluded based on the screening findings (1 had a solely financial motivation to participate, making it unadvisable to include this person under Dutch Good Research Practice, 1 was diagnosed with a first-degree atrioventricular block, 1 had TPO antibody positivity), and 27 individuals were included and went on to complete the T3 challenge study.

### B. Group Characteristics

Baseline characteristics of the study population are presented in Table 1. Our study population consisted of healthy, older middle-aged participants. The offspring (n = 14) and control (n = 13) groups were similar in terms of age, sex, and body mass index. Maternal age was higher in offspring than in controls (*P* < .01), confirming the longevity phenotype with respect to which offspring was selected for the LLS. The same trend was observed in paternal age, although this difference was not statistically significant (*P* = .06). Liver and kidney function were within normal range in all individuals and were similar in offspring and controls. All participants were clinically euthyroid at baseline, with higher TSH and similar fT4 levels in offspring and controls (TSH *P* = .05, fT4 *P* = .17). The fT3/fT4 ratio at baseline was higher in offspring than in controls (*P* = .005).

**Table 1.** Baseline characteristics of the 3,5,3′-triiodothyronine challenge study

	Offspring (n = 14)	Partners (n = 13)	*P*
Age of mother, y^*a*^	94 (91-97)	75 (70-87)	.003
Age of father, y^*a*^	94 (73-96)	78 (68-84)	.06
Men, n (%)	9 (64)	5 (36)	.18
Age, y	69 (67-72)	70 (66-72)	.63
BMI, kg/m^2^	25.5 (3.8)	26.2 (4.3)	.61
Weight, kg	79.0 (14.3)	78.3 (14.1)	.96
Height, cm	175.6 (10.2)	172.5 (9.0)	.54
Fat mass, kg	22.7 (6.3)	26.0 (9.8)	.31
Lean mass, kg^*a*^	59.7 (42.1-64.8)	52.3 (43.9-65.1)	.58
GFR, mL/min/1.73 m^2^,^*b*^	75.2 (13.5)	77.8 (8.2)	.56
AST, U/L^*a*^	21.7 (19.8-24.0)	23.0 (20.0-26.8)	.34
ALT, U/L	19.9 (5.8)	20.5 (5.0)	.80
TSH, mU/L	2.6 (1.2)	2.0 (1.1)	.05
fT4, pmol/L	14.3 (2.1)	15.1 (1.9)	.26
fT3, pmol/L	4.39 (0.36)	4.02 (0.49)	.03
fT3/fT4 ratio	0.313 (0.046)	0.267 (0.029)	.005

Data are shown as mean (SD) or as median and interquartile range.

Abbreviations: ALT, alanine transaminase; AST, aspartate transaminase; BMI, body mass index; fT4, free thyroxine; GFR, glomerular filtration rate; TSH, thyrotropin.

^*a*^Median (interquartile range).

^*b*^In offspring, based on 13 values because of 1 missing sample.

### C. Circulating 3,5,3′-Triiodothyronine (T3) and Free T3 Following T3 Administration

**Figure 2.**
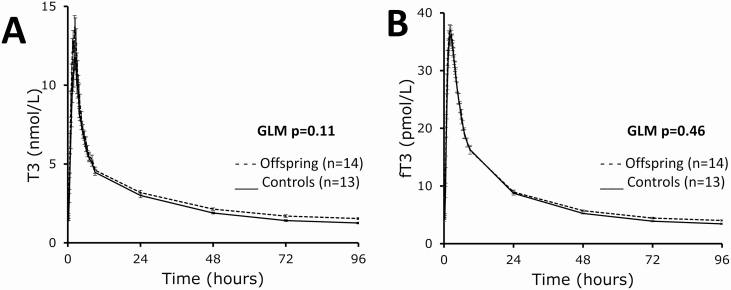
Five-day (96-hour) profile of mean circulating 3,5,3′-triiodothyronine (T3) and free T3 (fT3) in members of long-lived families, offspring (n = 14), and similarly aged controls, controls (n = 13) following a challenge with 100 µg T3 (oral administration). A, Whole-study (96-hour) mean circulating T3 profile as measured across 29 time points, general linear modeling (GLM): *P* = .11 between offspring and controls, *P* < .001 in time, *P* = .44 for time × group. B, Whole-study (96-hour) mean circulating fT3 profile as measured across 29 time points, GLM: *P* = .46 between offspring and controls, *P* < .0001 in time, *P* = 0.52 for time × group. Dashed lines: offspring, solid lines: controls. Error bars: SEM.

Following oral administration of 100 µg T3, circulating T3 and fT3 levels increased to supraphysiologic levels both in offspring and controls, reaching peak concentrations during study day 1 and subsequently decreasing in concentration and returning to baseline levels by study day 5 (Fig. 2A and 2B). The maximum concentration of T3 was not different between offspring and controls (mean [SEM] 12.5 [0.2] and 12.8 [0.2] nmol/L, respectively, *P* = .22), and the same was true for fT3 (mean [SEM] 37.6 [0.2] and 37.3 [0.4] pmol/L, respectively, *P* = .84). In both groups, the maximum concentration of T3 was reached 2 hours (median) following T3 administration (interquartile range [IQR] 1.75-2.00, *P* = .75 between offspring and controls), and the maximum concentration of fT3 was reached 2.25 hours (median) following T3 administration (IQR 2-2.30, *P* = .84 between offspring and controls). Circulating T3 and fT3 levels were similar between offspring and controls throughout the study (GLM *P* = .11 for T3, GLM *P* = .46 for fT3) and variable over time in both groups (GLM *P* < .001). There was no difference in the concentration profiles of T3 and fT3 over time between the 2 groups (GLM *P* = .44 for T3 and *P* = .52 for fT3). In addition, serum T3 concentrations after reaching maximal concentration were fitted to a biexponential function, y = a * e^–c * t^ + b * e^–d * t^. No differences between the kinetic parameters in the offspring and control group were present (Table 2).

**Table 2.** Kinetic parameters of exponential fitting the declining serum concentrations of 3,5,3′-triiodothyronine (T3) after administration of 100 µg T3

	Offspring	Partners	P
Coefficient α, nmol/L	8.82 ± 0.79	7.76 ± 0.45	.26
Coefficient β, nmol/L	4.60 ± 0.26	4.49 ± 0.33	.78
Slow rate constant, h^–1^	0.015 ± 0.001	0.016 ± 0.001	.47
Fast rate constant, h^–1^	0.984 ± 0.003	0.980 ± 0.003	.39

Data are shown as mean ± SEM. The formula used for the fitting was y = a * e^–c * t^ + b * e^–d * t^.

### D. Circulating Thyrotropin Following 3,5,3′-Triiodothyronine Administration

**Figure 3.**
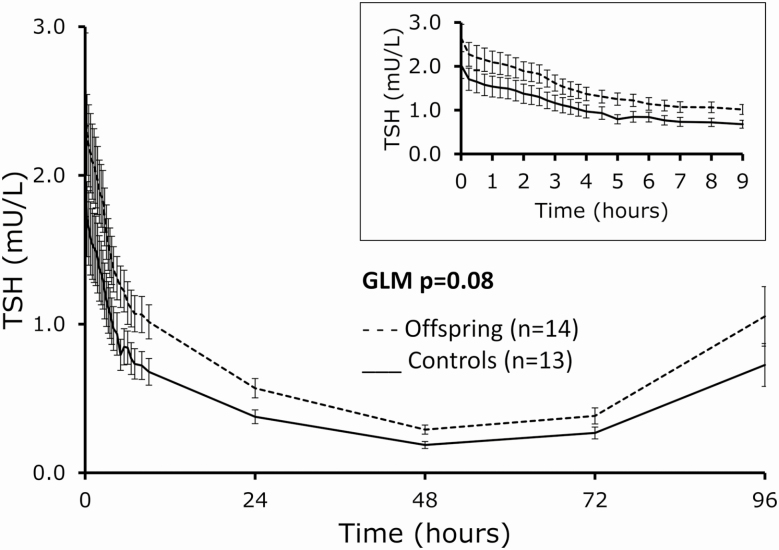
Five-day (96-hour) mean circulating thyrotropin (TSH) following a challenge with 100 µg 3,5,3′-triiodothyronine (T3, oral administration) in members of long-lived families, offspring (n = 14), and similarly-aged controls (n = 13), as measured across 29 time points. General linear modeling: *P* = 0.08 between offspring and controls. Inlay: detail of mean TSH profile during study day 1. Dashed lines: offspring, solid lines: controls. Error bars: SEM.

**Figure 4.**
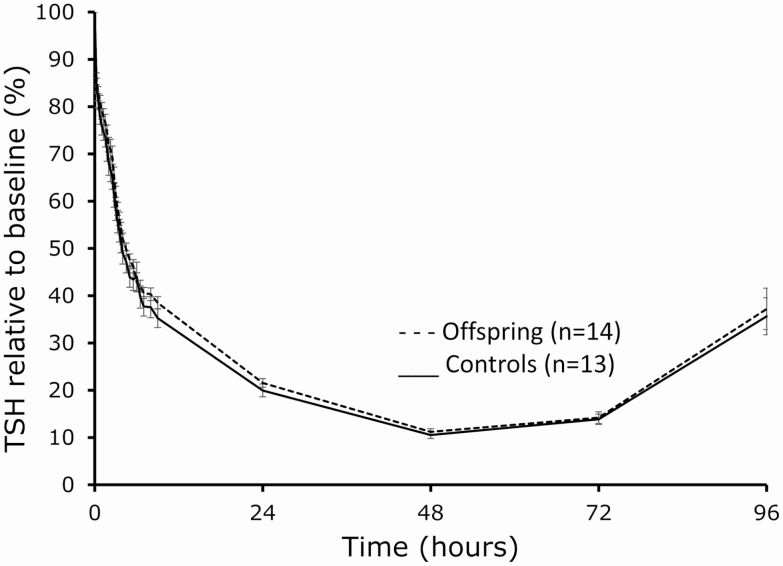
Five-day (96-h) profile of circulating thyrotropin (TSH), expressed as the percentage relative to baseline, following a challenge with 100 µg 3,5,3′-triiodothyronine (T3, oral administration) in members of long-lived families, offspring (n = 14), and similarly aged controls (n = 13), as measured across 29 time points. Dashed lines: offspring, solid lines: controls. Error bars: SEM.

Following T3 administration, TSH levels decreased in a biphasic manner with an acute decrease phase during study day 1 and a more gradual decrease on study days 2, 3, and 4, with levels returning to baseline by day 5 (Fig. 3). The nadir levels of TSH were similar between offspring and controls (mean [SEM] 0.281 [0.008] and 0.215 [0.012] mU/L, respectively, *P* = .19) and were reached around 48 hours (median) after T3 administration in both groups (IQR 48-48 hours in offspring and 48-72 hours in controls, *P* = .48). The 96-hour concentration profile of circulating TSH was similar between offspring and controls throughout the study (GLM *P* = .08 between offspring and controls). Baseline TSH levels between offspring and controls differed less than in the previous study (0.6 vs 0.8 mU/L) resulting in a borderline statistical difference (*P* = .05). We had previously observed that offspring have higher circulating TSH levels than controls as a part of their familial longevity phenotype. Therefore, we calculated the percentage decline in TSH throughout the study relative to baseline TSH to further account for any potential difference in TSH levels between offspring and controls. The relative TSH decline (%) was similar between offspring and controls throughout the study period (Fig. 4).

## 3. Discussion

In this study, we investigated 2 hypotheses related to the thyroid axis in familial longevity: whether familial longevity is associated with higher T3 turnover and/or lower negative T3 feedback on TSH. Following a challenge with 100 µg T3 orally, we found that T3 and fT3 concentration profiles, as well as relative TSH decline, were similar between members of long-lived families and controls.

This is the first time that a single challenge study with such a potent dose of T3 has been performed in an older middle-aged population. Circulating T3 and fT3 increased to supraphysiological levels in proportion to those reported in healthy young adults with other doses [13-15]. As expected, the 100-µg T3 dose did not cause any serious adverse events, and no suspected unexpected serious adverse reactions were reported in our study, despite the transient high circulating T3 and fT3 levels, indicating the safety of this dose in a single administration in healthy, euthyroid, older middle-aged individuals.

We based the dose of oral T3 on a previously published dose-response curve study that used doses ranging from 40 to 1000 µg T3 [13] in conjunction with power calculations regarding the required sample size for several doses. We chose the dose of 100 µg for multiple reasons. First, treatment with synthetic T3 might be associated with undesirable effects such as dizziness, headache, hot flashes, sweating, weight gain, and heart palpitations with irregular heartbeat. These side effects were not reported using a single dose of 100 µg T3 [13]. Additionally, using a 100-µg T3 dose, our power calculations indicated a sample size sufficient to assess the underlying hypothesis of the study, which was feasible within the given timeline and study design. Using lower doses would have required a higher sample size, which might have caused recruitment bottlenecks.

Throughout the study, we observed comparable concentration profiles of circulating T3 and fT3 in offspring and controls, indicating that T3 turnover is likely similar in these 2 groups, at least under challenge conditions. Additionally, the levels of T3 and fT3 recovered to baseline in both groups, corresponding to the trend previously observed in young adults [14, 15]. In fact, T3 was chosen as an intervention for this study on the basis of its relatively short half-life compared to the other thyroid hormone, T4. Previous studies in healthy individuals found that T3 levels increase and TSH levels decrease within a few hours following oral T3 administration [13, 15], and that the half-life of T3 is about 6 hours to 1 day [14, 16]. In contrast, T4 has a half-life of about 4 to 6 days [14, 16], making it a less-suitable candidate for a short-term clinical study.

We calculated the percentage decline of TSH relative to baseline to investigate the negative feedback on TSH following T3 administration in offspring and controls. Circulating TSH declined in a similar way in offspring and controls, consistent with the (biphasic) decline previously reported in the literature [14, 15]. Our findings also confirm the previously reported TSH-T3 relationship observed in members from long-lived families compared to controls: In physiological conditions members from long-lived families have a stronger temporal relationship between TSH and free T3 than controls, in the absence of differences in the negative feedback by thyroid hormones on TSH [17]. We confirmed this here by showing a comparable decline in TSH levels in the presence of similar circulating T3 and fT3 levels. However, under physiological conditions T4 rather than T3 is the feedback signal for the hypothalamic-pituitary-thyroid axis, acting on the pituitary thyrotrope and the hypothalamic tanycyte, involved in the secretion of thyrotropin-releasing hormone, by local conversion of T4 in T3 by deiodinase D2 [18]. The demonstration that exogenous T3, leading to relatively high serum T3 concentrations, inhibits TSH secretion to a similar extent in offspring and controls does not necessarily exclude dissimilarities in feedback by low-dose exogenous T4, and further experiments are indicated to confirm or refute this possibility.

Altogether, several findings provide clues regarding the mechanisms that might underlie the differences in thyroid axis (higher TSH levels in the absence of differences in thyroid hormones) between members of long-lived families and controls. We recently performed a challenge study with recombinant human TSH in a subpopulation of the LLS, showing that members of long-lived families have a lower thyroidal responsivity to TSH stimulation than similarly aged controls [4]. In this study, the findings point to similar T3 turnover and similar negative T3 feedback control of TSH secretion in offspring and controls. In concert, these findings indicate that higher circulating TSH in offspring is not a result of less negative feedback by T3 on the pituitary, or a peripheral effect caused by higher turnover of T3. Rather, the primary mechanism behind the thyroid phenotype previously observed in offspring seems to be in the thyroid gland, in which lower responsivity to TSH requires higher circulating TSH levels to produce adequate quantities of thyroid hormones, a condition resembling early autoimmune thyroid failure, leading to subclinical hypothyroidism, characterized by normal serum T4 and high normal serum TSH concentrations.

The fT3/fT4 ratio was significantly higher in offspring, and this change could be caused by decreased T3 clearance or increased deiodinase D2 activity in organs such as the liver, kidney, and muscle. The declining serum T3 levels following T3 maximum, as well as the kinetic parameters fitted by a biexponential model, were comparable in offspring and controls. Therefore, the increased ratio likely reflects increased deiodinase D2 activity. This finding was previously reported in 805 nonagenarians from the LLS and 259 nonagenarians from the Leiden 85+ study, in whom higher fT3/fT4 ratio (and higher fT3 and lower fT4) were associated with lower mortality rate, independent of familial longevity status [19]. Apparently, a higher ratio implies a healthier status, but at this time any mechanistic explanation is speculative. Our findings agree with a recent Italian study that showed that centenarians with a lower fT3/fT4 and higher fT4 levels have impaired functional status and increased mortality [20].

Interestingly, an alternative explanation was offered in the review by Franceschi et al [21]. Previously, in animal models, glucuronidase activity demonstrated in fecal content is indicative of enterohepatic circulation of iodothyronines via the gut microbiome, which allows reabsorption of native T3 following hydrolysis of conjugated forms of T3. The fraction of reabsorbed T3 that escapes liver extraction may reenter the general circulation and contribute to the systemic pool of T3 [22]. In previous studies on centenarians, a longevity-specific gut microbiome remodeling and signature were observed in conjunctions with subtle differences in thyroid status, suggesting an association between the two [21]. Based on these data the hypothesis can be raised that increased reabsorption of T3 by the gut microbiome may contribute to the observed higher T3/T4 ratio in this study and previously published studies on centenarians.

It is tempting to speculate that the high circulating levels of TSH in offspring somehow contribute to their longevity phenotype. TSH receptors have been found in bone and adipose tissue, as well as in the thymus and the brain [23-27]. The effects of TSH signaling on these tissues are not yet well understood; further research is necessary to increase our understanding of TSH’s extrathyroidal role in physiology and aging.

The thyroid status and function in the oldest old are complex and very heterogeneous. This applies in particular for the physiological thyroid age-related changes that occur in the absence of overt clinically relevant pathologies. Although previously regarded as simply detrimental, such changes are currently best conceptualized as part of the systemic, adaptive remodeling that helps humans survive in the last decades of life. As such, these changes are intimately linked to other age-related changes such as those occurring in the gut microbiome and immune system, making it difficult to disentangle specific and unidirectional cause-effect relationships [21].

Our study had several strengths. All morning samples were taken with the participants in a fasted state, and all the other samples (throughout study day 1) were taken between standardized meals, thereby minimizing variation in hormone levels due to gastrointestinal absorption or nutrition differences. The study consisted of a large number of time points across the study, thereby allowing detailed profiling of the T3, fT3, and TSH concentrations over time following the T3 challenge, allowing us to observe any subtle differences in concentration profiles indicating polymorphism of the thyroid hormone receptor, deiodination enzymes, or enzymes of the sulphating pathways. We are able to conclude with confidence that the T3 concentration profile and TSH decline are similar between offspring and controls because this was measured across 25 time points on study day 1 alone.

In conclusion, we found that, following a challenge with 100-µg T3, offspring from long-lived families had similar T3, fT3, and TSH 96-hour concentration profiles compared to similarly aged controls, indicating similar T3 turnover and similar negative T3 feedback on TSH. These findings, in combination with previously observed higher TSH levels and lower thyroid responsivity to TSH in members of long-lived families, suggest that the cause of high circulating TSH levels in familial longevity lies in the thyroid gland, although we cannot exclude concomitant dissimilar T4 feedback or increased T4 turnover. The role of TSH in the longevity phenotype remains a matter of speculation and requires further research.

## Data Availability

The data sets generated and analyzed during the present study are not publicly available but are available from the corresponding author on reasonable request.

## Abbreviations

AUC: area under the curve
fT3: free 3,5,3′-triiodothyronine
fT4: free thyroxine
GLM: general linear modeling
IQR: interquartile range
LLS: Leiden Longevity Study
T3: 3,5,3′-triiodothyronine
T4: thyroxine
Tg: thyroglobulin
TPO: thyroid peroxidase
TSH: thyrotropin

## Additional Information

***Disclosure Summary:*** The authors have nothing to disclose.
